# Rescue of the 1947 Zika Virus Prototype Strain with a Cytomegalovirus Promoter-Driven cDNA Clone

**DOI:** 10.1128/mSphere.00246-16

**Published:** 2016-09-28

**Authors:** Megan C. Schwarz, Marion Sourisseau, Michael M. Espino, Essanna S. Gray, Matthew T. Chambers, Domenico Tortorella, Matthew J. Evans

**Affiliations:** Department of Microbiology, Icahn School of Medicine at Mount Sinai, New York, New York, USA; University of Pittsburgh School of Medicine

**Keywords:** Zika virus, cell culture, flavivirus, infectious clones

## Abstract

The recent Zika virus (ZIKV) outbreak has been linked to severe pathogenesis. Here, we report the construction of a plasmid carrying a cytomegalovirus (CMV) promoter-expressed prototype 1947 Uganda MR766 ZIKV cDNA that can initiate infection following direct plasmid DNA transfection of mammalian cells. Incorporation of a synthetic intron in the nonstructural protein 1 (NS1) region of the ZIKV polyprotein reduced viral cDNA-associated toxicity in bacteria. High levels of infectious virus were produced following transfection of the plasmid bearing the wild-type MR766 ZIKV genome, but not one with a disruption to the viral nonstructural protein 5 (NS5) polymerase active site. Multicycle growth curve and plaque assay experiments indicated that the MR766 virus resulting from plasmid transfection exhibited growth characteristics that were more similar to its parental isolate than previously published 2010 Cambodia and 2015 Brazil cDNA-rescued ZIKV. This ZIKV infectious clone will be useful for investigating the genetic determinants of ZIKV infection and pathogenesis and should be amenable to construction of diverse infectious clones expressing reporter proteins and representing a range of ZIKV isolates.

**IMPORTANCE** The study of ZIKV, which has become increasingly important with the recent association of this virus with microcephaly and Guillain-Barré syndrome, would benefit from an efficient strategy to genetically manipulate the virus. This work describes a model system to produce infectious virus in cell culture. We created a plasmid carrying the prototype 1947 Uganda MR766 ZIKV genome that both was stable in bacteria and could produce high levels of infectious virus in mammalian cells through direct delivery of this DNA. Furthermore, growth properties of this rescued virus closely resembled those of the viral isolate from which it was derived. This model system will provide a simple and effective means to study how ZIKV genetics impact viral replication and pathogenesis.

## Abstract (precis)

The study of ZIKV, which has become increasingly important with the recent association of this virus with microcephaly and Guillain-Barré syndrome, would benefit from an efficient strategy to genetically manipulate the virus. This work describes a model system to produce infectious virus in cell culture. We created a plasmid carrying the prototype 1947 Uganda MR766 ZIKV genome that both was stable in bacteria and could produce high levels of infectious virus in mammalian cells through direct delivery of this DNA. Furthermore, growth properties of this rescued virus closely resembled those of the viral isolate from which it was derived. This model system will provide a simple and effective means to study how ZIKV genetics impact viral replication and pathogenesis.

## INTRODUCTION

Zika virus (ZIKV) is a member of the family *Flaviviridae* in the genus *Flavivirus*. The viral genome consists of a capped positive-strand RNA of approximately 11,800 nucleotides that carries a single open reading frame (1). The approximately 3,423 amino acid viral polyprotein is cleaved by host and viral proteases into structural proteins (capsid [C], premembrane [prM], and envelope [E]), which form the infectious virion, and nonstructural (NS) proteins (NS1, NS2A, NS2B, NS3, NS4A, NS4B, and NS5). The NS proteins participate in virion assembly, innate immune suppression, and formation of a cytoplasmic replication complex that replicates the viral genome through a negative-strand RNA intermediate.

ZIKV was first identified nearly 70 years ago (2) and has been progressively circulating in various parts of the world, including Africa and Asia. ZIKV emergence in South America in the last 2 years marked the first observation of a link between infections and severe forms of pathogenesis, including microcephaly and Guillain-Barré syndrome (3). It is not known if the diseases caused by recent infections are a result of changes to the virus that increase pathogenesis or are influenced by host polymorphisms and immune reactions. The African, Asian, and South American ZIKV isolates are closely related phylogenetically; the original 1947 strain, termed MR766 (accession no. HQ234498) (2), and a recent isolate identified in Puerto Rico, termed PRV2015 (accession no. KU501215) (4), share 96.9% amino acid identity. Thus, the characterization of sequence changes between these isolates that impact replication and disease should be reasonably straightforward. Such studies would be greatly empowered by the availability of a simple system to assay the functional consequences of altering viral sequences (i.e., a “reverse genetic” system).

For most positive-strand RNA viruses, where the genome directly encodes the proteins required for its replication, transfection of a permissive cell with viral RNA is sufficient to initiate infection that eventually results in the release of viral progeny. These genomes can be cloned into plasmids with elements required for *in vitro* transcription of the long viral RNA for transfection. Such an approach has only recently been used to rescue a 2010 Cambodia ZIKV isolate (5). Since flaviviruses have capped RNA genomes, the transcription step can be circumvented by transfecting a plasmid carrying the viral genome under the control of an RNA polymerase II promoter, with the correct 3′ end of the viral RNA being generated by a hepatitis D virus ribozyme (HDVr) (6, 7). The plasmid DNA is required only to initiate transcription of the first round of viral RNA, which can serve as both a translation and a replication template to initiate “infection” of transfected cells. Indeed, this is the approach that Tsetsarkin et al. recently used to rescue a 2015 Brazil ZIKV isolate (8).

Flavivirus sequences are notoriously difficult to propagate in bacteria, likely because the presence of cryptic bacterial promoters permits expression of viral proteins that result in bacterial toxicity (reviewed in references 9 and 10). Numerous strategies have been devised to limit and prevent bacterial death, including the use of bacteria that are more resistant to this toxicity, very-low-copy-number plasmids such that fewer viral translation products are produced, and alternative organisms such as yeast for plasmid propagation. Another strategy entails the separation of the viral genome into multiple plasmids, where each piece can be excised by restriction enzyme digestion and ligated into the full-length viral cDNA, which can then serve as the template for *in vitro* transcription. Here, we used an alternative strategy to stabilize the 1947 MR766 ZIKV genome (2) in bacteria that has been successfully used to stabilize other positive-sense RNA viruses, including transmissible gastroenteritis coronavirus and Japanese encephalitis virus (11, 12) and, more recently, the 2015 Brazil ZIKV genome (8). We identified the major region of the MR766 genome that induced toxicity in bacteria and then cloned this sequence with a synthetic intron insertion to interrupt viral translation in bacteria. This RNA was spliced in mammalian cells to recreate the authentic viral genome, which efficiently initiated infectious virus production.

## RESULTS

### Identification and stabilization of a bacterially toxic ZIKV cDNA region.

**FIG 1:**
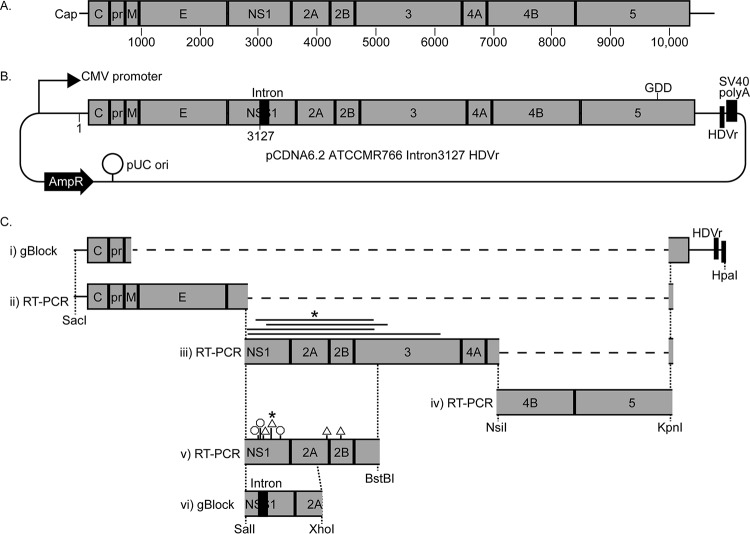
Illustrations of ZIKV genome and plasmid. (A) Organization of the ZIKV single-stranded RNA genome showing the 5′ cap and positions of the mature viral proteins inside the single open reading frame. (B) Organization of the pCDNA6.2 ATCCMR766 Intron3127 HDVr plasmid carrying the cDNA of the 1947 Uganda MR766 ZIKV genome under the transcriptional control of the CMV promoter. A ribozyme (HDVr) is positioned to trim the RNA to have the ZIKV 3′ end. An intron was inserted after nucleotide 3127 of the viral sequence. The positions of the “GDD” NS5 polymerase active site [changed to GNN to create a replication-incompetent Pol(−) plasmid], beta-lactamase resistance gene (AmpR), and origin of replication (pUC ori) are also marked. (C) Schematic representation of the steps (described in detail in Materials and Methods) used to create the above plasmid involved cloning synthetic DNA gBlocks or RT-PCR products derived from viral inoculum to progressively assemble the entire genome. Solid lines above step iii represent deletions, and octagons and triangles above step v represent the positions of nonsense and frameshift mutations, respectively, obtained when cloning each of these fragments. Asterisks indicate the mutant that was used in the next cloning step.

To generate a plasmid carrying the ZIKV cDNA (Fig. 1B), we purified RNA from MR766 inoculum and used reverse transcription-PCR (RT-PCR) to amplify overlapping regions of the viral genome, which were progressively cloned into the high-copy-number bacterial plasmid pCDNA6.2 (Fig. 1C). In this plasmid, the authentic 5′ end of the viral sequence was placed at the transcriptional initiation site of the cytomegalovirus (CMV) promoter, the simian virus 40 (SV40) polyadenylation site terminates transcription, and an HDVr trims the viral genome to its authentic 3′ end (Fig. 1B). The resulting transcript of the ZIKV genome is identical to that of a genome deposited in a host cell by a virion and is expected to initiate infection.

During cloning of the ZIKV RT-PCR products (scheme illustrated in Fig. 1C), we were unable to generate plasmids carrying an intact NS1 through NS3 coding sequence. Our first attempts at cloning this region of the ZIKV genome resulted in plasmids with large deletions, which we deduced to likely be a result of homologous recombination events by noting short stretches of sequence similarity at either side of the deleted segment. In an attempt to circumvent this issue, we screened a panel of bacterial strains with documented inefficiencies at homologous recombination. Indeed, using New England BioLabs’ Turbo competent cells allowed us to propagate a plasmid bearing the entire ZIKV cDNA. However, all such plasmids continued to lack an intact viral open reading frame due to the presence of either nonsense or frameshift mutations within the NS1 coding sequence. At this stage, we hypothesized that translation of viral polyprotein sequence from this region led to toxicity in bacteria. To test this hypothesis, we inserted a synthetic intron in NS1, such that the coding sequence would be disrupted in bacteria but splicing in mammalian cells would restore the viral RNA (Fig. 1B and C). Indeed, this plasmid could be stably propagated in bacteria, thus completing the generation of a plasmid containing the entire ZIKV cDNA. As a negative control for subsequent replication studies, we also generated a version of this plasmid encoding an inactivating GDD to GNN mutation in the viral NS5 RNA-dependent RNA polymerase (RDRP) catalytic active site [Pol(−)].

### Characterization of viral protein expression in ZIKV plasmid-transfected cells.

**FIG 2:**
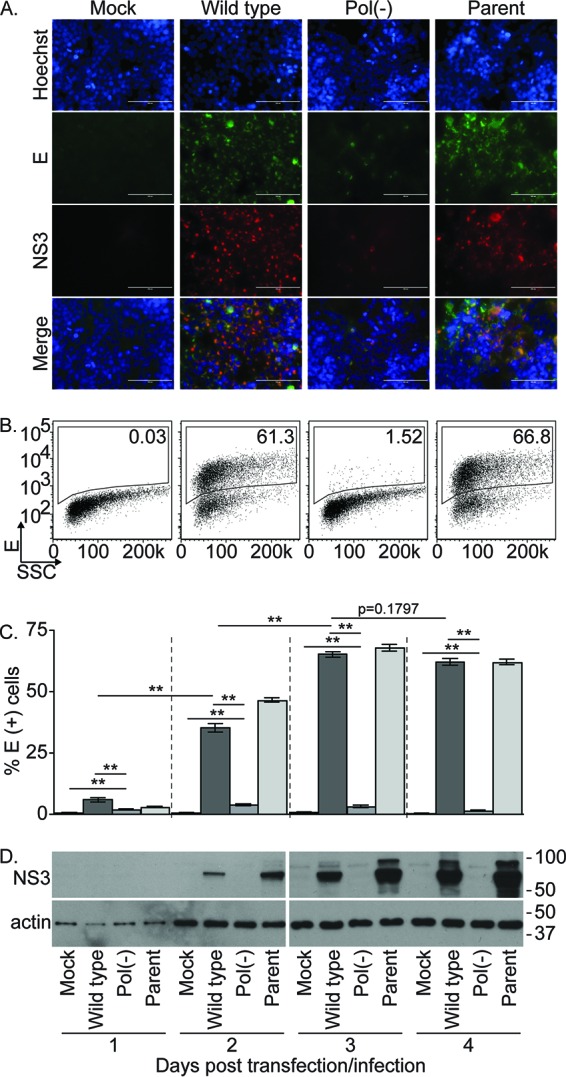
Characterization of cells transfected with a wild-type or Pol(−) MR766 ZIKV plasmid. (A) 293T cells were either mock treated, transfected with wild-type or Pol(−) plasmids, or infected with the parental MR766 virus. At 72 h posttreatment, these cells were immunostained for the ZIKV E (green) and NS3 (red) proteins and Hoechst counterstained (blue). (B) FACS analysis of transfected or infected 293T cells using a ZIKV E protein antibody at 72 h posttransfection or infection. Numbers in the top right corner indicate the percentage of cells expressing the E protein. SSC, side scatter. (C) Bar graph showing the percentage of 293T cells expressing the ZIKV E protein at 1 to 4 days posttransfection or infection, as determined by FACS analysis. Means and standard errors of the means from one of two representative experiments each performed in triplicate. *, *P* < 0.05; **, *P* < 0.005 (unpaired Student’s *t* test). (D) Immunoblot analysis for ZIKV was performed with an antibody against NS3 on 293T cells at 1 to 4 days after transfection or infection. Band sizes (kilodaltons) are indicated to the right of the blot.

To characterize the ability of the above plasmids to produce ZIKV proteins in mammalian cells, we transfected 293T cells with the wild-type and Pol(−) MR766 ZIKV plasmids and infected cells in parallel with the parental MR766 inoculum at an approximate multiplicity of infection (MOI) of 0.03 50% tissue culture infective dose (TCID_50_) per cell. Three days later, the cells were fixed and immunostained with antibodies against the ZIKV E and NS3 proteins (Fig. 2A). While all transfected and infected cells expressed both viral proteins, cells transfected with the wild-type MR766 plasmid exhibited larger amounts of antibody staining than those transfected with the Pol(−) plasmid. This difference was confirmed by quantifying E protein immunostaining by fluorescence-activated cell sorting (FACS) analysis (Fig. 2B). To examine viral protein expression over time, cells collected at various time points following transfection or infection were analyzed by FACS for E protein antibody staining or by immunoblotting of cell lysates with NS3 antibodies (Fig. 2C and D, respectively). While the percentage of E protein-positive cells increased over 3 days following transfection with the wild-type MR766 plasmid and infection with the parental MR766 virus, the E protein-positive Pol(−) plasmid-transfected cells remained below 5% throughout this time course (Fig. 2C). NS3 protein levels also increased over time in both wild-type plasmid-transfected and parental virus-infected cell populations and yet remained below the limit of detection in Pol(−) plasmid transfected cells. These results confirmed that although both the wild-type and Pol(−) MR766 ZIKV plasmids were able to express viral proteins, protein levels were higher and were detected in a greater percentage of cells transfected with the wild-type plasmid, which may be due to viral RNA replication and viral spread.

### Splicing occurs in cells transfected with a ZIKV plasmid.

**FIG 3:**
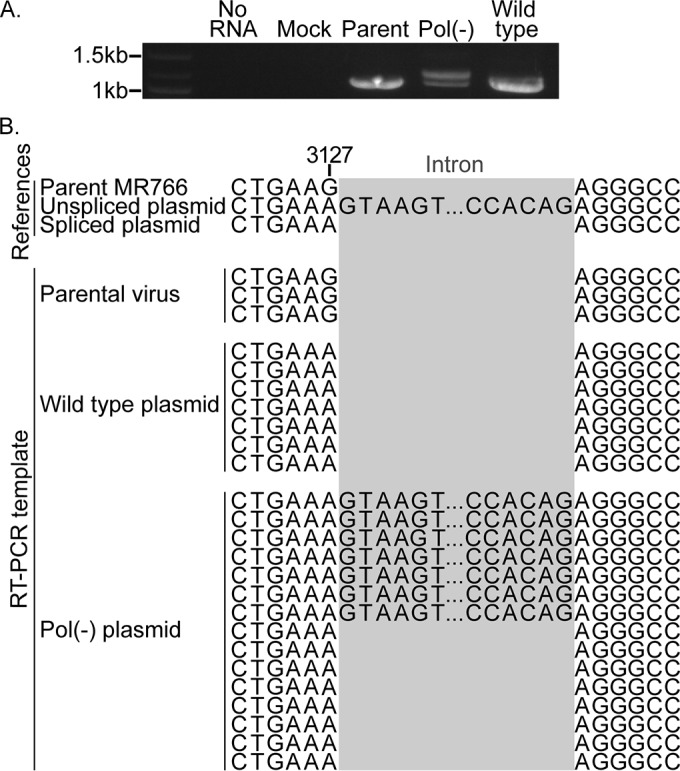
Confirmation of spliced RNA in cells transfected with a wild-type or Pol(−) MR766 ZIKV plasmid. (A) RT-PCR products from RNA from cells transfected with the wild-type or Pol(−) MR766 ZIKV plasmid or infected with the parental MR766 virus. Band sizes (kilobases) are indicated to the left of the gel. Two product sizes were observed, representing the unspliced (1,083-bp) and spliced (1,216-bp) template. (B) Sequence results of RT-PCR products from transfected or infected cells aligned to three reference sequences: parental MR766 virus, unspliced plasmid, and spliced plasmid.

Expression of NS3 in cells transfected with either the wild-type or the Pol(−) MR766 plasmid, as observed above (Fig. 2A), would require splicing of the viral RNA transcript. To directly examine this, total RNA was harvested 2 days following transfection with either plasmid, or infection with the parental MR766 virus, and RT-PCR was performed using oligonucleotides flanking the region of the intron-containing NS1 gene. As shown in Fig. 3A, a single DNA product was amplified from wild-type plasmid transfected cells that was similar in size to the parental MR766 RT-PCR product. This DNA fragment likely represented spliced NS1 RNA. However, two distinct RT-PCR products were observed from Pol(−) plasmid transfected cells, likely representing both spliced and unspliced RNA [Fig. 3A, “Pol(−)”].

Indeed, sequence analysis of bacterial plasmid clones of these RT-PCRs demonstrated that all products from wild-type plasmid-transfected cell RNA lacked the inserted intron (Fig. 3B). In contrast to parental virus RT-PCR products (Fig. 3B), these sequences carried a single silent G3127A mutation that was inserted during intron cloning, which indicated that these RNAs were generated from the MR766 plasmid. While spliced sequences were observed in eight of the 15 bacterial plasmid clones of the Pol(−) RT-PCR products, seven of these clones retained the intron sequence (Fig. 3B). These results confirmed that splicing can remove the intron from RNA transcribed from both the wild-type and Pol(−) MR766 plasmids. Furthermore, the greater abundance of spliced RNA in wild-type plasmid-transfected cells may represent amplification due to viral RNA replication.

### Cells transfected with the wild-type MR766 plasmid produce infectious virus.

**FIG 4:**
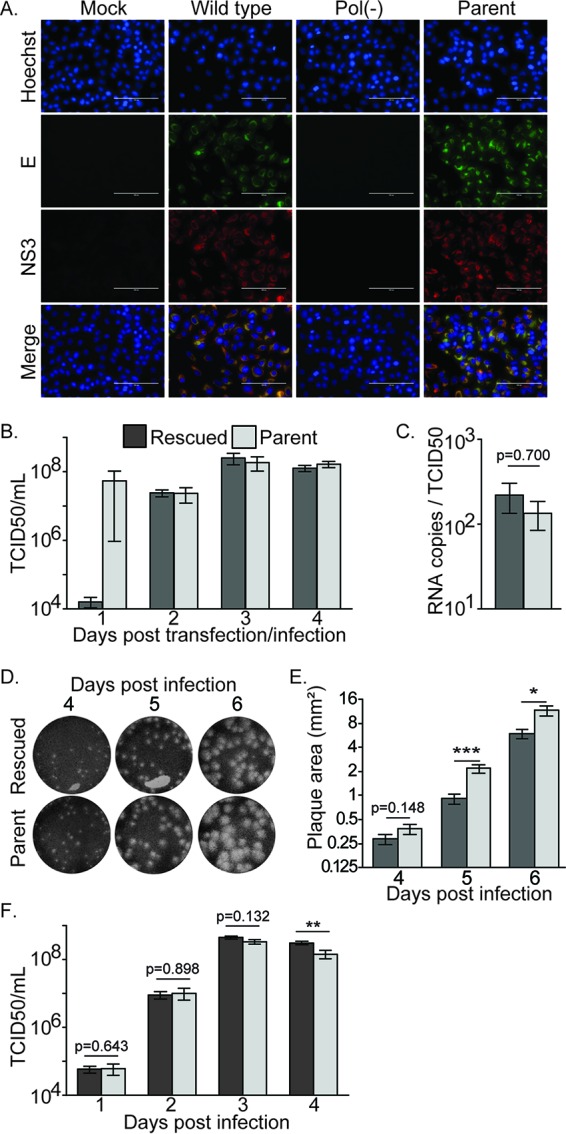
Characterization of virus rescued from a wild-type or Pol(−) MR766 ZIKV plasmid in comparison to the parental MR766 virus. (A) At 72 h postinfection, rescued wild-type, Pol(−), parental MR766, or mock-infected Vero cells were immunostained for the ZIKV E (green) and NS3 (red) proteins and Hoechst counterstained (blue). (B) The relative titers of virus produced from 293T cells at 1 to 4 days after transfection with the wild-type or Pol(−) plasmid or infection with the parental MR766 virus or a mock control were determined by Vero cell TCID_50_ assay based on ZIKV-associated CPE, expressed in TCID_50_ per milliliter. Means and standard errors of the means from 3 independent experiments each performed in triplicate. (C) Specific infectivity of the wild-type rescued or parental MR766 was determined by calculating the RNA copies per TCID_50_ based on values produced from qRT-PCR of supernatant from infected Vero cells as well as titers determined by Vero cell TCID_50_ assay. Means and standard errors of the means from 3 independently passaged virus supernatants. (D) Representative images of Vero cell plaque assays with wild-type rescued and parental MR766 viruses fixed and stained at the indicated days postinfection. (E) Graph of plaque sizes (in square millimeters) quantified using ImageJ software. Means and standard errors of the means from over 30 independent plaques for each virus and time point. (F) A growth curve was performed to compare the growth curve kinetics of the wild-type rescued and parental MR766 viruses. Vero cell infections were performed for 1 h at an MOI of 0.01, and supernatant was harvested and titers were determined at 1 to 4 days postinfection by Vero cell TCID_50_ assay. Values of titers are expressed in TCID_50_ per milliliter. Means and standard errors of the means from 3 independent experiments each performed in triplicate. *, *P* < 0.05; **, *P* < 0.005; ***, *P* < 0.0005 (unpaired Student’s *t* test).

To evaluate production of infectious virus, we challenged Vero cells with supernatant from 293T cells transfected with the wild-type or Pol(−) plasmid or infected with the parental MR766 virus at an approximate MOI of 0.03 TCID_50_ per cell. The Vero cells were immunostained with E and NS3 antibodies 3 days postchallenge (Fig. 4A). The addition of supernatants from wild-type plasmid-transfected or parental virus-infected 293T cells resulted in readily detectable levels of viral proteins. Conversely, no staining was observed in Vero cells challenged with Pol(−) plasmid transfected 293T cell supernatant. Thus, cells transfected with the wild-type MR766 plasmid produced infectious particles in a manner that was dependent on the capacity of the viral RNA to replicate.

Infectious virus from transfected and infected 293T cells was quantified by endpoint dilution assay on Vero cells (TCID_50_) by measuring ZIKV-associated cytopathic effect (CPE). As observed above, 293T cells transfected with the Pol(−) MR766 plasmid failed to release detectable levels of infectious virus (data not shown). At 24 h after transfection with the wild-type MR766 plasmid, 293T cells produced 1.6 × 10^4^ TCID_50_/ml, which increased to 2.4 × 10^7^ and 2.5 × 10^8^ TCID_50_/ml at 2 and 3 days posttransfection, respectively (Fig. 4B). Parallel infection of 293T cells with the parent MR766 virus resulted in supernatants containing between 2.6 × 10^7^ and 2.2 × 10^8^ TCID_50_/ml. However, it should be noted that the 24 h time point likely contains residual input virus, as we chose to conduct overnight infections and did not wash input virus away prior to collecting the first time point. Nevertheless, comparing the kinetics of virus released following plasmid transfection with that following virus infection may not be relevant because RNA delivered in a virion can be directly translated and replicated while plasmid-expressed RNA requires transcription, splicing, and export from the nucleus.

### Comparison of the growth properties of rescued and parental MR766 viruses.

The previously developed systems for producing infectious 2010 Cambodia ZIKV and 2015 Brazil ZIKV from plasmids showed that the rescued viruses were attenuated in comparison to the parental inoculum (5, 8). To compare the fitness levels of our parental and rescued MR766 viruses, we first calculated their Vero cell specific infectivities. To ensure that only virion-associated RNA was quantified, the rescued virus was initially passaged twice at an MOI of 0.1 in Vero cells. The rescued and parental MR766 viruses exhibited similar and not significantly different specific infectivity values of 218 and 130 RNA molecules per TCID_50_ (Fig. 4C). To directly compare growth properties of the 293T cell-rescued MR766 virus to those of the parental inoculum using a strategy similar to that used on previously published systems, we performed plaque assays (Fig. 4D and E). When visualized at 4 days postinfection, the rescued and parental MR766 ZIKV-induced plaques exhibited a 1.35-fold, but not statistically significant, difference. According to the work of Tsetsarkin et al., the rescued 2015 Brazil ZIKV isolate made plaques that were 2.5-fold smaller than the parental virus plaques at this time point (8). A retrospective analysis of plaque sizes in the publication by Shan et al. showed that their rescued 2010 Cambodia ZIKV produced plaques that were approximately 3.3-fold smaller than parental virus plaques at 4 days postinfection (5). However, we were able to detect slight attenuation of our rescued virus, as we observed statistically significant 2.4- and 2-fold differences in rescued and parental MR766 ZIKV plaque sizes when we stained our plaque assays at 5 and 6 days postinfection, respectively (Fig. 4D and E).

To further compare the growth properties of the rescued and the parent viruses, multicycle growth curve experiments were performed in which Vero cells were infected (MOI of 0.01) for 1 h with either the rescued wild-type virus or the parental virus, followed by several washes to remove input virus. Supernatants were collected and filtered daily up to 4 days postinfection, and then titers of the supernatants were determined by Vero cell TCID_50_ assay. The results of the growth curve revealed that our rescued virus has growth kinetics comparable to those of the parental virus (Fig. 4F). Growth curves initiated at a range of MOIs also demonstrated comparable growth kinetics between the rescued and parental viruses (data not shown). These results are in stark contrast to previously published multicycle growth curve results with the 2010 Cambodia and 2015 Brazil ZIKV rescued viruses, which exhibited approximately 10- to 50-fold slower spread than their respective parental isolates. Therefore, although extended plaque assays indicate that our rescued MR766 virus may exhibit some attenuation in comparison to the parental MR766 isolate, its replication characteristics are closer to those of its parental isolate than to the 2010 Cambodia and 2015 Brazil ZIKV rescued viruses.

## DISCUSSION

In this work, we describe the creation of a plasmid-based rescue system for the prototype Uganda 1947 MR766 ZIKV. Other groups have published ZIKV infectious virus rescue systems. The first such system was a clone of the 2010 Cambodia ZIKV isolate cDNA (5). Here, a T7 RNA polymerase promoter directed *in vitro* transcription of the viral RNA for subsequent transfection into permissive host cells for initiation of infection. Our approach to express the ZIKV RNA from an RNA polymerase II promoter with a ribozyme positioned at the 3′ end to trim the viral transcript allowed us to avoid the potentially arduous *in vitro* transcription step and directly transfect the plasmid into host cells. The Shan et al. system lessened the toxicity of ZIKV cDNA in bacteria by using a very-low-copy-number bacterial plasmid. In our system, the deleterious sequence within the genome was disrupted by the addition of an intron, which allowed the successful amplification of this sequence in a high-copy-number plasmid (Fig. 1). In a more recent publication, Tsetsarkin et al. described their derivation of a similar plasmid-based rescue system for a 2015 Brazil ZIKV isolate (8). They also found that the toxicity of the ZIKV cDNA could be reduced by insertion of introns in the viral open reading frame.

A second recently published ZIKV rescue system avoided bacterial toxicity by transfection of overlapping synthetic DNA fragments carrying the MR766 cDNA (13). In permissive host cells, these fragments were joined by recombination to launch viral RNA transcription from a CMV promoter, analogous to our approach. Although not reported, it is likely that the efficiency of recombination limited the initial rescued virus titers. Thus, generating high-titer virus stocks in this system required amplification by passage of rescued virus. Our plasmid-based system allowed the production of high-titer virus directly from transfected cells, which results in an increased capacity to generate isogenic virus stocks. This would be particularly important when analyzing unstable viral genomes, including genomes that are naturally attenuated in specific host cells or those carrying mutations that limit viral replication and thus may revert during passage.

Virus produced from the previously published 2010 Cambodia and 2015 Brazil ZIKV isolate rescue systems exhibited slower growth kinetics than the parental viruses, potentially due to sequence diversity within the parental virus populations that provided a fitness advantage (5, 8). Conversely, our rescued MR766 virus grew identically to the parental isolate in Vero cell multicycle growth curve experiments and produced similarly sized plaques in standard 4-day infection plaque assays. Thus, even though we observed plaque size differences between our parental and rescued virus in extended infection, and thus likely more sensitive plaque assays, we believe that our MR766 rescued virus is less attenuated than the rescued 2010 Cambodia and 2015 Brazil ZIKV. It is unlikely that the methods of rescue (transfection of *in vitro* transcribed RNA versus plasmid DNA) impacted virus attenuation. Instead, we hypothesize that viral genetics influenced postrescue attenuation, and this may be greatly influenced by the passage history of a viral isolate. The 1947 MR766 isolate may not require the same sequence diversity as the 2010 Cambodia and 2015 Brazil ZIKV isolates to reach optimal fitness in Vero cells. The MR766 virus has been subjected to more rounds of amplification in Vero cells than these more recent strains, which may have preadapted it to efficient cell culture replication. Ultimately, rescued virus attenuation should not be a serious complication to experiments to study viral determinants of replication or pathogenesis. Researchers using any ZIKV rescue system would merely need to monitor the stability of engineered sequences of interest and compare phenotypes between wild-type and mutant viruses rescued in parallel.

Overall, the plasmid-based rescue system described in this work is a simple and elegant system that paves the way for a variety of future ZIKV experiments. Reporter viruses can be created by cloning fluorescent or bioluminescent proteins into the MR766 plasmid as an efficient strategy to quantify and monitor infections. This system will also permit the modification of viral sequences to study how genetic determinants impact the viral life cycle and pathogenesis. We will also derive analogous plasmids bearing the cDNA sequence of other ZIKV isolates, including those currently circulating in the Americas. Such systems will allow the detailed comparison of the molecular determinants for potential strain-specific ZIKV replication and pathogenesis mechanisms and/or efficiencies.

## MATERIALS AND METHODS

### Cell lines and culture.

293T (provided by Charles M. Rice, Rockefeller University) and Vero (ATCC, Manassas, VA) cells were grown as previously described (14) in Dulbecco’s modified Eagle’s medium (DMEM; Gibco BRL Life Technologies, Gaithersburg, MD) with 100 U/ml penicillin, 100 µg/ml antibiotic-antimycotic (Gibco BRL Life Technologies, Gaithersburg, MD), and 10% fetal bovine serum (FBS; Gibco BRL Life Technologies, Gaithersburg, MD).

### ZIKV stock propagation and infectious virus quantification.

The ATCC VR-84 1947 Uganda MR766 ZIKV isolate (provided by Adolfo García-Sastre, Icahn School of Medicine at Mount Sinai) was propagated in Vero cells. Virus titers were determined by CPE-based limited dilution assay on Vero cells, based on techniques previously described (15). Here, 1 × 10^4^ cells were seeded in each well of a 96-well plate the day before being infected with 100 µl of virus serially diluted in DMEM with 2% FBS (typically 6 to 8 wells per dilution). Six days postinfection, infected wells were scored by evidence of ZIKV-induced CPE and the TCID_50_ was calculated according to the method of Reed and Muench (16).

### Plasmid construction.

The pWNII-green fluorescent protein (GFP) subgenomic West Nile virus (WNV) replicon plasmid was used as a scaffold for construction of a ZIKV cDNA clone (provided by Theodore Pierson, NIH) (7). The WNV cDNA sequence was replaced with the MR766 ZIKV cDNA sequence such that the 5′ and 3′ termini of each virus were precisely swapped. Several MR766 sequences have been deposited in GenBank. We sequenced our ATCC MR766 isolate and found that it was identical to GenBank accession file HQ234498 (17). We based our plasmid off this sequence to be assured of working with an infectious virus sequence and to be able to directly compare growth characteristics of rescued and parental viruses in subsequent experiments. However, HQ234498 does not include the full 5′ and 3′ MR766 untranslated regions (UTRs). Thus, we based these regions of our clone off the original AY632535 MR766 sequence with one exception: sequencing our MR766 virus showed an additional C nucleotide in the 3′ UTR after nucleotide 10696 of this sequence (to read ATTGACCGTGGGA). All nucleotide positions referred to in this work are relative to this sequence, comprised of the HQ234498 sequence with AY632535 5′ and 3′ UTR extensions and the additional C nucleotide in the 3′ UTR. Our final plasmid carried five silent mutations in the MR766 cDNA: A1213G (creating a StuI restriction site), T1840C, C2491T, G3127A, and C9133T.

We used a multistage cloning scheme to construct our MR766 plasmid (illustrated in Fig. 1C). We first obtained a synthetic DNA, gBlock (Integrated DNA Technologies, Coralville, IA), comprised of 15 nucleotides of CMV promoter sequence before the SacI site, nucleotides 1 to 874 and 10033 to 10807 of the MR766 cDNA, the HDVr, and the first half of the SV40 polyadenylation sequence up to 15 nucleotides past the HpaI site. This gBlock was cloned using the In-Fusion kit (Clontech, Mountain View, CA) into the SacI and HpaI sites of pWNII-GFP, replacing the WNV sequence with MR766 sequence and adding unique restriction sites to fill in the missing internal viral sequence (Fig. 1C, step i). An RT-PCR product spanning the SacI site of the CMV promoter through nucleotides 1 to 2926 was amplified with oligonucleotides ME-O-1751 (5′ GGTCTATATAAGCAGAGCTCGTTTAGTGAACCGAGTTGTTGATCTGTGTGAGTCAGACTGCGACAGTTCG) and ME-O-1787 (5′ TGGTTCTCCCAGTTGGTACCTCAAGCGGACATTCCTTC) and In-Fusion cloned into the SacI and KpnI sites of the above acceptor plasmid, which resulted in a plasmid missing the ZIKV nucleotides 2926 to 10033 (Fig. 1C, step ii). Attempts to In-Fusion clone (SalI to KpnI) a PCR product spanning the MR766 SalI-to-NsiI fragment, generated with ME-O-1775 (5′ CAGTTTTGTTGTCGACGGTGACACACTGAAGGAATGTCCGCTTGAGCACAGAG) and ME-O-1782 (5′ TGGTTCTCCCAGTTGGTACCATGGTGGCGGCATAATAG), generated plasmids with deletions of more than 1 kb within the SalI-to-BstBI region, suggesting that this region contained sequences that were toxic in bacteria (Fig. 1C, step iii). A clone with a deletion from nucleotides 3074 to 5052 was chosen to proceed with cloning of the remaining MR766 3′ regions. This section was amplified with oligonucleotides ME-O-1778 (5′ AGGGATGCCATTTTATGCATGGGACCTTGGAGTCCCGCTGCTAATG) and ME-O-1770 (5′ TGGTTCTCCCAGTTGGTACCCAGTCAAC), and the resulting product was In-Fusion cloned into the NsiI and KpnI sites of the above plasmid, resulting in a plasmid missing only nucleotides 3074 to 5052 of the MR766 cDNA (Fig. 1C, step iv). All prior plasmids were propagated in Stellar competent cells (Clontech, Mountain View, CA). The deletions within the SalI-to-BstBI region appeared to occur at regions of repeated sequence, suggesting that they were created by recombination. Thus, we screened alternative strains of bacteria for ones that were less prone to this phenomenon by attempting to In-Fusion clone a SalI-to-BstBI PCR product, generated with oligonucleotides ME-O-1775 and ME-O-1813 (5′ AGCATCGAGGGTTCGAAACATTCAACCG) (Fig. 1B, step v). NEB Turbo competent *Escherichia coli* (New England BioLabs, Ipswich, MA) permitted propagation of clones bearing this entire region, thus completing the MR766 cDNA. These bacteria were also grown at 30°C for all cloning and amplification steps, including recovery following heat shock transformation. However, all derived clones carried nonsense or frameshift mutations, suggesting that the ZIKV open reading frame still induced toxicity in these bacteria. We chose a plasmid with a frameshift mutation created by a single G nucleotide insertion after nucleotide position 3170 for further cloning. A gBlock bearing 15 nucleotides before and after the SalI and XhoI sites, respectively, was synthesized with a silent G3127A mutation in the MR766 coding sequence followed immediately by the synthetic intron from plasmid pHTN (Promega, Madison, WI) (GenBank accession no. JF920304), which is based on the human beta-globin intron. In-Fusion cloning of this DNA into the above plasmid resulted in a plasmid that was stably propagated in bacteria. We termed this plasmid pCDNA6.2 ATCCMR766 Intron3127 HDVr.

We also generated a version of this plasmid that carries an inactivating GDD-to-GNN mutation in the viral NS5 RNA-dependent RNA polymerase (RDRP). We generated this plasmid by amplifying pCDNA6.2 MR766 Intron3127 HDVr with oligonucleotides carrying two nucleotide coding mutations in the NS5 RDRP active site as well as a silent mutation to create an SphI restriction site, ME-O-1917 (5′ CTATCATCGATTGGCTTCACAACGCAGTTATTTCCACTGACCGCC) and ME-O-1918 (5′ GTTGTGAAGCCAATCGATGATAGGTTTGCGCATGCCCTCAGGTTC). This PCR product was amplified with ME-O-1779 (5′ GCAAGCGGCCACGCGTCTGCACCAAAGAAGAG) and ME-O-1770 to clone into the MluI and KpnI sites. We termed this plasmid pCDNA6.2 MR766 Intron3127 Pol(−) HDVr.

### 293T cell transfection and supernatant collection.

Plasmid-based ZIKV was produced in 293T cells by transfections performed in triplicate. One day prior to transfection, 5 × 10^5^ cells/well were seeded in 12-well polylysine-coated plates. Cells were transfected with 0.2 µg of DNA per well with 40 µl of Opti-MEM (Gibco BRL Life Technologies, Gaithersburg, MD) and 3 µl of TransIT (Mirus Bio, Madison, WI) that had been incubated with the DNA for 20 to 60 min prior to transfection. Supernatants from transfected cells were collected 1 to 4 days posttransfection and filtered (0.45 µm pore size).

### qRT-PCR.

RNA was extracted from 140 µl cell supernatant using the QIAamp viral RNA minikit (Qiagen, Valencia, CA) and eluted in 60 µl of buffer AVE (Qiagen) after on-column DNase treatment with RNase-free DNase (Qiagen). For quantifying ZIKV RNA, quantitative reverse transcriptase PCR (qRT-PCR) was performed on 2 µl of cell supernatant RNA extracted using ZIKV-specific primers (5′ TTGGTCATGATACTGCTGATTGC and 5′ CCYTCCACRAAGTCYCTATTGC) and probe (5′ 6-carboxyfluorescein [FAM]-CGGCATACAGYATCAGGTGCATWGGAG-minor groove binder nonfluorescent quencher [MGB-NFQ]) (Thermo Fisher Scientific, Waltham, MA) and the LightCycler 480 Master hydrolysis probe kit (Roche Applied Science, Indianapolis, IN) using the LightCycler 480 II real-time PCR system (Roche Applied Science). Sequences of the primers and probe targeting ZIKV have been modified from previously published sequences (18). Quantification of ZIKV RNA copies per milliliter of supernatant was performed against a standard curve of *in vitro*-transcribed MR766 ZIKV RNA.

### Immunoblot analysis.

Immunoblot analysis was performed as previously described (19) with primary antibodies against NS3 (rabbit polyclonal antiserum 459, raised against a peptide representing amino acids 456 to 469 of the GenBank AAV34151 MR766 sequence) and actin (AC-15; Sigma-Aldrich, St. Louis, MO), horseradish peroxidase (HRP)-conjugated anti-rabbit and anti-mouse secondary antibodies (Thermo Fisher Scientific, Waltham, MA), and Immobilon chemiluminescent HRP detection reagent (EMD Millipore, Billerica, MA).

### Immunofluorescence.

Cells were fixed with 4% paraformaldehyde and stained with the rabbit polyclonal antiserum 459 against ZIKV NS3 or a primary antibody targeting the flavivirus E proteins (clone D1-4G2-4-15; EMD Millipore) and a goat anti-rabbit or a goat anti-mouse secondary antibody conjugated to Alexa Fluor 568 and 488 (Thermo Fisher Scientific, Waltham, MA), respectively, using methods previously described (14). The nucleus was visualized with Hoechst stain (Thermo Fisher Scientific, Waltham, MA). Images were acquired with the Evos microscope (Thermo Fisher Scientific, Waltham, MA).

### Flow cytometry.

Cells were fixed with 4% paraformaldehyde and stained with the primary 4G2 flavivirus E antibody (EMD Millipore, Billerica, MA) and a goat anti-mouse secondary antibody conjugated to Alexa Fluor 647 (Thermo Fisher Scientific, Waltham, MA), using methods previously described (20). Cells were analyzed using an LSRII flow cytometer (Becton, Dickinson, Franklin Lakes, NJ).

### RT-PCR analysis of splicing.

RNA was extracted from cells 48 h after transfection with the wild-type MR766 plasmid or Pol(−) plasmid or infection with the parental MR766 virus or a mock control using the PureLink RNA minikit (Thermo Fisher Scientific, Waltham, MA). The extracted RNA was used as a template for random hexamer-primed cDNA synthesis using the SuperScript III First-Strand synthesis system (Thermo Fisher Scientific, Waltham, MA). Five hundred nanograms of cDNA was used for PCR using the Expand High-Fidelity PCR system (Roche Life Sciences, Indianapolis, IN) with oligonucleotides flanking the region into which the intron was cloned: ME-O-1722 (ATGTCCGCTTGAGCACAGAG) and ME-O-1738 (AGCGATGTTGTCAGTGCGTG). PCR products were ligated into pGEM-T vector (Promega, Madison, WI) for subsequent propagation in bacteria and sequencing.

### Growth curves.

Vero cells (2 × 10^6^) were seeded in a 10-cm petri dish, 1 day prior to infection, in triplicate, and then infected at a multiplicity of infection (MOI) of 0.01 Vero cell TCID_50_ per cell in DMEM with 2% FBS. Virus was removed 1 h later, and cells were washed twice with PBS before 6 ml of DMEM was added with 2% FBS. Supernatants were collected and filtered (0.45 µm pore size) daily for the next 4 days. Supernatant infectivity was assayed as previously described.

### Plaque assays.

One day prior to infection, 7.5 × 10^5^ Vero cells were seeded in polylysine-coated 12-well plates in DMEM with 2% FBS. For infection, medium was replaced with 300 µl of 10-fold serial dilutions (with a starting MOI of 5) of either the parental or rescued virus. After 2 h at 37°C, 1.5 ml 0.8% methylcellulose in DMEM with 2% FBS was added to each well. At 4 to 6 days postinfection, cells were fixed in 1 ml 4% paraformaldehyde for 1 h at room temperature. Cells were washed twice with PBS and stained with 500 µl of 0.5% crystal violet solution in ethanol. Plates were washed twice with water and air dried. Plates were scanned, and plaque sizes were measured using ImageJ software (National Institutes of Health, Bethesda, MD; http://imagej.nih.gov/ij/).

### Statistical analysis.

Data were analyzed using an unpaired Student *t* test (*, *P* < 0.05; **, *P* < 0.005; ***, *P* < 0.0005) on Prism software (GraphPad Software). Values in graphs represent the mean and standard error from experiments performed in triplicate or quadruplicate, with between two and five independent experiments.

### Accession number(s).

The sequence of the MR766 ZIKV genome with the additional C nucleotide in the 3′ UTR was deposited in GenBank under accession number KX830960. The sequence for plasmid pCDNA6.2 ATCCMR766 Intron3127 HDVr has been deposited in GenBank under accession number KX830961.
